# Telehealth Satisfaction in Patients Receiving Virtual Atrial Fibrillation Care: Quantitative Exploratory Study

**DOI:** 10.2196/50232

**Published:** 2023-09-14

**Authors:** Kathy L Rush, Lindsay Burton, Cherisse L Seaton, Peter Loewen, Brian P O'Connor, Lana Moroz, Kendra Corman, Mindy A Smith, Jason G Andrade

**Affiliations:** 1 School of Nursing University of British Columbia, Okanagan Kelowna, BC Canada; 2 Faculty of Pharmaceutical Sciences University of British Columbia Vancouver, BC Canada; 3 Centre for Cardiovascular Innovation Department of Medicine University of British Columbia Vancouver, BC Canada; 4 Department of Psychology University of British Columbia, Okanagan Kelowna, BC Canada; 5 The Cardiac Atrial Fibrillation Specialty Clinic Vancouver General Hospital Vancouver, BC Canada; 6 Department of Family Medicine Michigan State University East Lansing, MI United States; 7 Montreal Heart Institute Université de Montréal Montréal, BC Canada

**Keywords:** atrial fibrillation, telehealth, telehealth satisfaction, self-efficacy, attitudes toward technology, attitude, attitudes, satisfaction, telemedicine, cardiology, heart, adoption, eHealth, perception, perceptions

## Abstract

**Background:**

Telehealth can optimize access to specialty care for patients with atrial fibrillation (AF). Virtual AF care, however, may not fit with the complex needs of patients with AF.

**Objective:**

This study aims to explore the correlation among attitudes toward health care technologies, self-efficacy, and telehealth satisfaction as part of the future planning of virtual AF clinic care.

**Methods:**

Patients with AF older than 18 years from an urban-based, highly specialized AF clinic who had an upcoming telehealth visit were invited to participate in a web-based survey. The survey asked about demographic characteristics; use of technology; general, computer, and health care technology self-efficacy (HTSE) and health care technology attitudes, using a validated 30-item tool; and telehealth satisfaction questionnaire using a validated 14-item questionnaire. Data were analyzed with descriptive statistics, correlational analyses, and linear regression modeling.

**Results:**

Participants (n=195 of 579 invited, for a 34% response rate) were primarily older, male, and White, had postsecondary schooling or more, and had high self-reported overall and mental health ratings. A variety of technologies were used in their daily lives and for health care, with the majority of technologies comprising desktop and laptop computers, smartphones, and tablets. Self-efficacy and telehealth satisfaction questionnaire scores were high overall, with male participants having higher general self-efficacy, computer self-efficacy, HTSE, and technology attitude scores. After controlling for age and sex, only HTSE was significantly related to individuals’ attitudes toward health care technology. Both general self-efficacy and attitude toward health care technology were positively related to telehealth satisfaction.

**Conclusions:**

Consistent with a previous study, only HTSE significantly influenced attitudes toward health care technology. This finding confirms that, in this regard, self-efficacy is not a general perception but is domain specific. Considering participants’ predominant use of the telephone for virtual care, it follows that general self-efficacy and attitude toward health care technology were significant contributors to telehealth satisfaction. Given our patients’ frequent use of technology and high computer self-efficacy and HTSE scores, the use of video for telehealth appointments could be supported.

## Introduction

Atrial fibrillation (AF) is the most common sustained cardiac arrhythmia, affecting 1%-2% of the general population and increasing significantly with age, affecting 12% of those 80 years and older [1,2]. Virtual care using telehealth services optimizes access to care for patients with AF receiving care at specialty clinics, often located in urban centers [3]. Telehealth is defined as remote clinical care involving the exchange of information required for accurate diagnosis, treatment, and care continuity and may be either synchronous or asynchronous [4-6]. With its rapid emergence during the COVID-19 pandemic to promote care continuity while ensuring patient and clinician safety [7], virtual care’s many advantages, including convenience, improved access, and efficiency, have prompted efforts to sustain its routine integration into patient care [8]. However, the chronic, progressive, and unpredictable nature of AF may make virtual care challenging for this population. A recent study found that patients receiving virtual AF clinic care did not always experience virtual care as a fit with their needs and concerns and questioned the quality of their care [3].

User satisfaction with telehealth, an important indicator of health care quality, has become a key to telehealth success [9]. In the few applications of virtual care to arrhythmia or AF clinic care, patients’ satisfaction with telehealth has ranged from 70% to 98% [10-12]. Little is known about the factors contributing to variation in patients’ satisfaction with virtual AF clinic care. Two factors that may play an important role in patients’ telehealth satisfaction are their attitudes toward technology and their confidence or self-efficacy in using technology. However, to date, there is a paucity of evidence exploring their role. This is a significant gap in the virtual care research for patients with AF and limits future planning of virtual AF clinic care to serve this population best.

Several studies have evaluated the use of telehealth to increase self-efficacy for chronic disease self-management [13], but less is known about the impact of self-efficacy on satisfaction with telehealth. Additionally, there is limited empirical evidence regarding attitudes toward technology among patients with AF. Koshy et al [14] assessed the attitudes of patients with arrhythmias (primarily AF or atrial flutter) toward self-monitoring mobile or wearable technology and found approximately 70% were interested in the technology but reported its complexity as a limiting factor, a finding that may reflect low self-efficacy. Similarly, a qualitative study of perceptions and attitudes of patients with AF toward e-tool self-care technology found that patients’ reluctance was related to unfamiliarity with the technology; lack of ownership of certain technology (smartphone and tablet); perceptions of e-tools being complicated, impractical, and difficult to learn; and literacy challenges. This evidence suggests that attitudes toward technology in patients with AF are directly related to their lack of confidence or low self-efficacy in using it.

Rahman et al [15] identified 3 self-efficacy factors that were important for shaping an individual’s attitude toward health care technologies—general, computer, and health technology self-efficacy (HTSE). In their study of graduate and undergraduate students, only HTSE positively influenced attitudes toward the use of health technologies. Both general and computer self-efficacy positively influenced HTSE, but neither influenced individuals’ attitudes toward using health care technologies. This indicates that targeting more situation-specific self-efficacy in a younger and likely healthier population could enhance the uptake and satisfaction of these technologies. It is unclear if this holds true for older populations and those with chronic diseases, such as AF, who may be more familiar with health care technologies but less confident in their computer skills.

**Figure 1:**
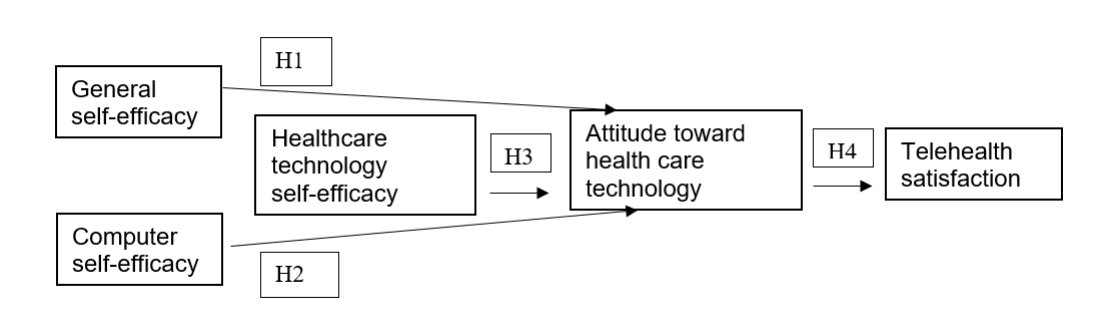
Conceptual model based on Rahman et al [15]. H: hypothesis.

In this study, we explored the relationship between attitudes toward health care technologies, self-efficacy, and telehealth satisfaction as part of future planning for virtual AF clinic care. We adapted the conceptual model by Rahman et al [15] to explore the influences on telehealth satisfaction due to our sample’s exclusive use of telehealth, older age, and potentially lower self-efficacy with computers. This study addressed the following hypotheses (Figure 1):

H1: Participants’ general self-efficacy positively influences their attitudes toward health care technology use.

H2: Participants’ computer self-efficacy positively influences their attitudes toward health care technology use.

H3: Participants’ health technology self-efficacy positively influences their attitudes toward health care technology use.

H4: Participants’ attitudes toward health care technology use positively influences their telehealth satisfaction.

## Methods

### Study Design

This study used a web-based cross-sectional survey to explore influences on patient satisfaction with telehealth received from a specialty AF clinic during the COVID-19 pandemic. This study was conducted in partnership with an urban-based, highly specialized AF clinic in western Canada.

### Ethics Approval

Participants provided informed consent digitally prior to completing the survey. Participants also consented to release appointment dates with the AF clinic to the research team. The study received ethics approval from the university research ethics board (H19-03601).

### Sample and Recruitment

Patients older than 18 years with an AF diagnosis who spoke and understood English or who had a family member who could assist were eligible to participate*.* Recruitment was open from November 2020 to September 2021. The clinic’s booking clerk sent a letter, by regular mail or email, to all patients with upcoming clinic appointments during the recruitment period. The letter detailed the ongoing research study and informed patients to expect a telephone call from a research team member to discuss their eligibility or interest in the study. The clinic shared patient contact information with the research team using secure file transfer. Subsequently, a research assistant (a physician or a licensed practical nurse) who had no prior relationship with participants contacted patients by telephone. Patients who agreed to participate were emailed a link to the web-based consent form and survey. Patients who completed the survey were entered into a random draw to win 1 of 3 CAD $150 (US $118.50) gift certificates.

### Data Collection

Data were collected using a web-based survey hosted on Qualtrics (Qualtrics International Inc). The survey took approximately 30 minutes to complete. Individuals who wanted assistance, had an unreliable internet connection, or had no smartphone or computer access were given the option to complete the survey over the phone with a research assistant. The booking clerk extracted the AF clinic appointment dates of participants from the AF clinic electronic medical record and shared them with the research team using a secure file transfer.

### Measures

#### Sociodemographic Characteristics and Health Status

Questions were asked with regard to age, sex, marital status, race or ethnicity, education, and income.

#### Technology

Questions were asked with regard to what types of technology the participants used for daily life and health care (eg, appointments and information) as well as the type and cost of internet service they used. Participants were also asked to rate their satisfaction with internet services on a scale from 1 (poor) to 10 (excellent) on reliability, speed, support, security, and availability.

#### Self-Efficacy and Health Care Technology Attitudes

We used a validated 30-item tool that captures general self-efficacy, computer self-efficacy, HTSE, and attitude toward health care technology [15]. Items are scored on a 7-point Likert scale ranging from 1 (strongly disagree) to 7 (strongly agree). Scores range from 1 (low self-efficacy or attitude) to 7 (high self-efficacy or positive attitude).

#### Telehealth Satisfaction Questionnaire

The telehealth satisfaction questionnaire (TSQ) [16] is a validated 14-item 5-point Likert scale tool to measure patient satisfaction with telehealth. Participants responded to items on a scale ranging from “strongly disagree” (1) to “strongly agree” (5). Scores ranged from 1 (low satisfaction) to 5 (high satisfaction). Overall satisfaction is calculated as the mean of all 14 items; subscales include the quality of care provided (8 items), similarity to face-to-face interaction (5 items), and perception of the interaction (1 item). Participants were asked to consider their appointments with the AF clinic when answering the TSQ items. Item 4, “I can see my health care provider as if we met in person,” was removed from our analyses due to the high number of telephone appointments, making the item irrelevant to our population. The overall TSQ Cronbach α in our sample with this item removed was .898.

### Data Cleaning

Less than 5% of data were missing for each of the key study variables, but data were not missing completely at random (Little missing completely at random [MCAR] *P*=.003). Patients missing more than a third of the scale data (n=8) were removed from the analysis. Patients missing values on less than a third of the scales were replaced with multiple imputations (n=8).

### Analysis

SPSS (version 28; IBM Corp) was used to conduct all analyses. Descriptive statistics were used to describe the patient characteristics and sociodemographics. Our analysis was guided by the conceptual model for users’ attitudes toward health care technology use [15]. A multivariate analysis of variance was used to evaluate the differences among male participants and female participants, and correlations were used to examine relationships with age on participant characteristics and the self-efficacy scales, attitude toward health care technology, and telehealth satisfaction. A linear regression model to address H1 to H3 was conducted with attitude toward health care technology as the dependent variable using age and sex as control variables, and general self-efficacy, computer self-efficacy, and HTSE as predictor variables. A second linear regression model was conducted to address H4 with telehealth satisfaction as the dependent variable using age and sex as control variables and attitude toward health care technology, general self-efficacy, computer self-efficacy, and HTSE as predictor variables.

Normality was examined using histograms and P-P plots. Telehealth satisfaction and general self-efficacy were slightly negatively skewed but considered acceptable given the sample size. Two participants with low telehealth satisfaction scores consistently came up as influential cases using standard techniques for handling outliers; thus, we opted to Windsorize their telehealth satisfaction scores to .01 less than the next lowest score of 1.83, allowing their responses to be retained in the analyses. Both regression analyses met assumptions of linearity, heteroscedasticity, and multicollinearity. *P* values less than .05 were considered statistically significant.

## Results

### Descriptive Results

A total of 579 patients were eligible for inclusion and contacted during the recruitment period; 352 (55% response rate for invited patients) were sent the web-based survey invitation, and 195 completed the survey (34% response rate for eligible participants). Participants were an average age of 65.36 (range 33-91 years, SD 10.32) years, were primarily male (n=122, 62.5%), White (n=175, 89.7%), and had postsecondary schooling or more (n=129, 66.2%). Participants had a high self-reported rating of health, with 72.3% (n=141) of participants rating their overall health as good or excellent and 87.7% (n=171) of participants rating their mental health as good or excellent (Table 1). The appointment modality used at the time of recruitment was almost exclusively telephone (n=177, 90.8%).

**Table 1:** Demographic characteristics.

Characteristics			Participants (n=195), mean (SD)		Male (n=122), mean (SD)		Female (n=73), mean (SD)		*P* value^a^
Age (years)			65.4 (10.3)		63.4 (9.9)		68.7 (10.1)		<.001
**Marital status**									.16
	Single (never married)	15 (7.7)		9 (7.4)		6 (8.2)			
	Divorced, separated, or widowed	32 (16.4)		15 (12.3)		17 (23.3)			
	Married, remarried, or common law	146 (74.9)		96 (78.7)		50 (68.5)			
	Missing	2 (1.0)		2 (1.6)		N/A^b^			
**Ethnicity**									.50
	Caucasian	175 (89.7)		109 (89.3)		66 (90.4)			
	Other	17 (8.7)		12 (9.8)		5 (6.8)			
	Missing	3 (1.5)		1 (0.8)		2 (2.7)			
**Education**									.97
	Graduate or professional degree	12 (6.2)		7 (5.7)		5 (6.8)			
	Postsecondary training or degree	117 (60.0)		74 (60.7)		43 (58.9)			
	Some postsecondary	36 (18.5)		23 (18.9)		13 (17.8)			
	High school or less	30 (15.4)		18 (14.8)		12 (16.4)			
**Income (US $)**									<.001
	<$25,000	13 (6.7)		5 (4.1)		8 (11.4)			
	$25,000-$50,000	38 (19.5)		16 (13.1)		22 (30.1)			
	$51,000-$75,000	40 (20.5)		18 (14.8)		22 (30.1)			
	Over $75,000	99 (50.8)		81 (66.4)		18 (24.7)			
	Missing	5 (2.6)		2 (1.6)		3 (4.1)			
**Housing**									.18
	Apartment or condominium	53 (27.2)		30 (24.6)		23 (30.1)			
	Own detached home	127 (65.1)		85 (69.7)		42 (57.5)			
	Other	15 (7.7)		7 (5.7)		8 (11.0)			
	Missing	1 (0.5)		N/A		1 (1.4)			
**Living situation**									.03
	Live alone	37 (19.0)		18 (14.8)		19 (26.0)			
	Live with partner	117 (60.0)		75 (61.5)		42 (57.5)			
	Live with others	39 (32.0)		29 (23.8)		10 (13.7)			
	Missing	2 (1.0)		N/A		2 (2.7)			
**First clinic appointment**									.47
	Within past month	42 (21.5)		23 (18.9)		19 (26.0)			
	1 month to 6 months ago	28 (14.4)		19 (15.6)		9 (12.3)			
	6 months to 1 year ago	26 (13.3)		18 (14.8)		8 (11.0)			
	1 year to 2 years ago	25 (12.8)		13 (10.7)		12 (16.4)			
	Over 2 years ago	60 (30.8)		40 (32.8)		20 (27.4)			
	Missing	14 (7.2)		9 (7.4)		5 (6.8)			
**First appointment relative to the COVID-19 pandemic**									.92
	Prior to	86 (44.1)		54 (44.3)		32 (43.8)			
	After declaration	95 (48.7)		59 (48.4)		36 (49.3)			
	Missing	14 (7.2)		9 (7.4)		5 (6.8)			
**Appointment modality at time of recruitment**									.21
	Telephone	177 (90.8)		110 (90.2)		67 (91.8)			
	Video	11 (5.6)		9 (7.4)		2 (2.7)			
	In-person	1 (0.5)		1 (0.8)		0 (0)			
	Missing	6 (3.1)		2 (1.6)		4 (5.5)			

^a^Wilcoxon rank sum test; Pearson chi-square test; Fisher exact test.

^b^N/A: not applicable.

Participants used a variety of technologies in their daily lives and for health care, with the majority of technologies comprising desktop and laptop computers, smartphones, and tablets (Table 2). On average, participants had a high rating of their internet service on availability (8.6/10, SD 1.5), reliability (8.4/10, SD 1.5), security (8.0/10, SD 1.8), speed (8.1/10, SD 1.6), and support (7.34/10, SD 2.2).

**Table 2:** Technology-related characteristics of participants.

Technology-related characteristics		Participants (n=195), n (%)
**Technology (daily life)**		
	Desktop computer	100 (51.3)
	Laptop computer	129 (66.2)
	Smartphone	169 (86.7)
	Tablet	105 (53.8)
	e-Reader	2 (1)
	Landline or nonsmartphone	3 (1)
	Smartwatch	4 (2)
**Technology (health care)**		
	Desktop computer	76 (39)
	Laptop computer	90 (46)
	Smartphone	112 (57.4)
	Smartphone/tablet apps	28 (14)
	Tablet	43 (22)
	Smartwatch/Fitbit	29 (15)
	Heart or blood pressure–related device	3 (1)
	Landline or nonsmartphone	10 (5)
**Internet type**		
	Cable	94 (48)
	Fiber optic	93 (48)
	Satellite	2 (1)
	Dial-up	1 (0)
	Missing	5 (3)
**Internet cost per month (US $)**		
	$10-$50	19 (10)
	$51-$100	91 (47)
	$101-$150	51 (26)
	More than $150	19 (10)
	Internet is included in rent/housing payments	2 (1)
	Unknown	6 (3)
	No internet	3 (1)
	Missing	3 (1)

General self-efficacy, computer self-efficacy, HTSE, technology attitude scale scores, and TSQ scores are shown in Table 3. On average, self-efficacy scores were high overall (mean >5 on a scale from 1 to 7), as were the TSQ scores (mean 4.16, SD 0.73 on a scale from 1 to 5). Male participants reported higher general self-efficacy, computer self-efficacy scores, HTSE, and technology attitude scores. There was a small negative correlation between age and computer self-efficacy (*r*=−.265, *P*<.001) and HTSE (*r*=−.248, *P*<.001). Participants’ telehealth satisfaction, general self-efficacy, and technology attitude were not correlated with age.

**Table 3:** Self-efficacy, technology attitude scale, and telehealth satisfaction questionnaire scores^a^ of participants.

Self-efficacy	Participants (n=195), mean (SD)	Male (n=122), mean (SD)	Female (n=73), mean (SD)	*F* test (*df*)	*P* value
General self-efficacy	5.89 (0.77)	5.98 (0.78)	5.74 (0.74)	4.54 (1)	.03
Computer self-efficacy	5.38 (1.27)	5.63 (1.17)	4.95 (1.31)	14.52 (1)	<.001
Health technology self-efficacy	5.63 (1.01)	5.81 (0.93)	5.32 (1.08)	11.24 (1)	<.001
Technology attitude	5.46 (0.87)	5.66 (0.80)	5.13 (0.88)	18.81 (1)	<.001
TSQ^b^ score	4.16 (0.73)	4.21 (0.70)	4.08 (0.78)	1.38 (1)	.24

^a^Multivariate analysis of variance results indicated a statistically significant difference between male participants and female participants on the following combined dependent variables: telehealth satisfaction, computer self-efficacy, HTSE, and technology attitude (*F*_5,189_=5.302, *P*<.001; Wilk Λ=0.877; partial η^2^=0.123).

^b^TSQ: telehealth satisfaction questionnaire.

### Hypotheses 1 to 3—Predictors of Attitude Toward Health Care Technology

After controlling for age and sex, when entered in a regression simultaneously with general and computer self-efficacy, only HTSE was significantly related to individuals’ attitudes toward health care technology (see Table 4). Thus, hypotheses 1 and 2 are refuted, and hypothesis 3 is supported. Among the control variables, sex was related to attitude toward health care technology (see positive β −.29, *P*<.001). Exploring this with an independent *t* test, male participants had a more positive attitude (mean 5.66, SD 0.80) compared to female participants (mean 5.13, SD 0.88; 2-tailed t_193_=4.34; *P*<.001). We explored separate regression models for male participants and female participants, and these followed a similar pattern, so the overall model is presented.

**Table 4:** Regression examining the association between attitude toward health care technology (outcome; overall R2=0.38; F5,194=22.66; model *P*<.001) and the following predictors: age, sex, general self-efficacy, computer self-efficacy, and health care technology self-efficacy.

Predictors			β		Coefficient *P* value
**Control variables**					
	Age	−.04		.61	
	Sex^a^	−.29		<.001	
**Independent variables**					
	General self-efficacy	.10		.12	
	Computer self-efficacy	−.16		.06	
	HTSE^b^	.62		<.001	

^a^Dummy variable: 0=male, 1=female; standardized β coefficients are reported.

^b^HTSE: health care technology self-efficacy.

### Hypothesis 4—Predictors of Telehealth Satisfaction

After controlling for age and sex, both general self-efficacy and attitude toward health care technology were positively related (whereas computer self-efficacy and HTSE were unrelated) to telehealth satisfaction when entered in a regression simultaneously (see Table 5), thus supporting hypothesis 4 and adding the dimension of general self-efficacy. The same pattern of results was found for the 3 subscales of telehealth satisfaction, except general self-efficacy did not significantly predict the perception of the interaction subscale.

**Table 5:** Regressions examining the association between telehealth satisfaction scale scores (outcomes; overall R2=0.29; F6,194=12.74; model *P*<.001) and the following predictors: age, sex, general self-efficacy, computer self-efficacy, health care technology self-efficacy, and attitude toward health care technology.

Predictors		β	Coefficient *P* value
**Control variables**			
	Age	.02	.77
	Sex^a^	−.09	.23
**Independent variables**			
	General self-efficacy	.24	<.001
	Computer self-efficacy	−.16	.01
	HTSE^b^	.02	.85
	Attitude toward health care technology	0.47	<.001

^a^Dummy variable: 0=male, 1=female; standardized β coefficients are reported; separate regressions for each telehealth satisfaction subscale were conducted.

^b^HTSE: health care technology self-efficacy.

## Discussion

### Principal Findings

This study offers valuable insights into the role of attitudes toward technology and self-efficacy on telehealth satisfaction among patients with AF receiving specialty AF clinic care. Our participants had high use of smartphones and computers in their daily lives, with moderate general self-efficacy, computer self-efficacy, and HTSE despite the majority of AF clinic appointments being conducted using the telephone. Similar to Rahman et al [15], only HTSE was significantly related to individuals’ attitudes toward health care technology. Although male participants reported higher self-efficacy and technology attitude scores, the overall model was similar for male participants and female participants. Both general self-efficacy and attitudes toward health care technology were related to telehealth satisfaction.

### Self-Efficacy and Attitude Toward Health Care Technology

This is the first study to examine the predictive role of self-efficacy on attitudes toward technology among patients receiving virtual AF care. Self-efficacy has consistently been shown to be a significant predictor of attitudes toward technology [15]. Current findings suggested that only domain-specific HTSE positively influenced participants’ attitudes toward health care technology use, whereas general self-efficacy and computer self-efficacy did not. Thus, hypotheses 1 and 2 were not supported; this finding is consistent with Rahman et al [15], who found no significant influence of general self-efficacy and computer self-efficacy on attitude toward health care technology use in undergraduate and graduate students. Our findings confirm Bandura’s [17] extensive work that self-efficacy is not a general perception but is domain specific, and it should vary across situations and be tailored to the domain of interest. Provincial efforts to expand and encourage provider adoption of both virtual visits, and patient portals could serve as a means of increasing self-efficacy and indirectly support the use of other forms of health care technology. While reports on patient portals have not specifically addressed HTSE, studies show improved patient engagement and patient-provider communication through the use of portals [18].

Overall findings did not differ between male participants and female participants, although male participants had more positive attitudes toward technology than female participants. Male participants’ more positive attitudes toward technology use resonate with findings from a meta-analysis of gender and attitudes toward technology use in nonpatient populations. Cai et al [19] found a continuing sex attitudinal gap, with male participants showing more favorable attitudes toward technology use than female participants. However, the gender gap was smaller when the general attitude was differentiated between dimensions (affect, belief, self-efficacy, and mixed) for affect and self-efficacy but not belief. The attitude scale used in this study was specific to health care technology, and future research could explore possible attitude dimensions.

### Predictors of Telehealth Satisfaction

Previous studies have suggested that self-efficacy is an influential factor in predicting intention to use telehealth services [20,21]. Given the constraints of COVID-19 policies and limitations of in-person service, the reality of care has become telehealth as the default service. Patients, by necessity, are using telehealth services; yet few studies have explored the drivers of patient satisfaction. Studies that do explore telehealth satisfaction have been limited by how it is measured. One systematic review found telehealth satisfaction was measured inconsistently and often adapted for each unique setting, making comparisons across studies challenging [22].

Our findings extend this work by exploring self-efficacy and telehealth attitudes as a predictor of telehealth satisfaction. We found that attitudes toward health care technology and general self-efficacy significantly positively influenced telehealth satisfaction. Similarly, a 2014 study exploring interest in telehealth among patients with a raised risk for cardiovascular disease found that higher technology confidence and positive perceptions of telehealth were associated with greater interest in using telehealth [23]. They also found that telehealth modality-specific context predicted interest in that modality but not others. For instance, confidence in computers predicted interest in using computers for telehealth [23]. Due to our participants’ predominant use of the telephone, it logically follows that general self-efficacy would be the more significant contributor to telehealth satisfaction over computer self-efficacy and HTSE.

Even with the telephone as the leading modality of telehealth appointments, study participants were high technology users, with overall positive technology attitudes and moderate technology-specific self-efficacy. Nearly all participants reported using a smartphone in daily life. However, far fewer reported using the smartphone for health care. The moderate self-efficacy and regular use of various technologies indicate that our participants have the capacity to use telehealth modalities beyond just the telephone. Evidence suggests the advantages of using video over the telephone for telehealth appointments [24], yet the telephone continues to outpace the use of video in telehealth appointments [25]. Although a recent study identified a lack of confidence in using technology as a leading challenge faced by participants using telehealth services [26], given the advantages of video-supported care and the findings of this study, patients should be offered this option.

Although virtual AF care systems have advanced efficiencies for patients in terms of access, convenience, cost savings, and encounter time to discuss risk factor modification [3,27] greater efficiencies are needed as virtual AF management telehealth systems and services continue to evolve and expand as a complementary format to in-person care. Providing additional virtual care options for patients such as email, text messaging, and a patient portal is not a simple task and would require access to high-speed internet, training for both patients and providers, and optimizing office workflow through reassigning tasks [28-31]. The use of wearable medical devices that transfer data electronically, such as electrocardiogram and blood pressure monitors, may increase self-efficacy through their use but will also require systems to support best practice and integration of data into patient medical records [32,33]. Further development of user-friendly virtual technologies, as well as training and orientation to the technology and clinical workflows, is needed to implement virtual care models and promote patient and clinician adoption [27] and, in turn, increase HTSE.

### Strengths and Limitations

This study provides a novel investigation into predictors of telehealth satisfaction among patients with AF receiving virtual care. The use of a standardized multidimensional telehealth satisfaction scale provided a more fulsome interpretation of the full range of the construct. However, it limits comparison to other studies of telehealth satisfaction in this population, which used either a 1-item global satisfaction measure [11] or a multi-item (n=6) nonstandardized measure [12]. Our sample had more male participants, who were significantly younger than the female participants, consistent with the demographic of patients with AF. However, we controlled for age and sex in the regression models to mitigate potential influences on our findings. There is the possibility of selection bias, with more positive telehealth users completing the survey. Predominant AF clinic use of the telephone modality limits the generalizability of findings. Further exploration of the effects of computer self-efficacy and HTSE in a sample that used video and telephone modalities would be desirable. Further research could also explore how these findings might generalize to other patient populations and could examine relationships in specific patient populations with other conditions (eg, cancer and diabetes) comorbid with AF. Indeed, the management of comorbid AF is a major challenge for clinicians and patients [34], but finding solutions to optimize management is imperative since evidence indicates that multimorbidity in association with AF, though common, is associated with increased all-cause mortality [34]. There is considerable potential for virtual care systems to address and improve the management of multimorbidity. This includes addressing issues such as prioritization, coordination, and management of multiple diseases [35]. Because patients with multiple diseases often have multiple appointments, with potentially competing treatment goals, nonintegrated care services, and multiple guidelines, virtual care has the potential to alleviate these issues and streamline care [35].

### Conclusions

Patients with AF receiving virtual specialty care predominantly by telephone had overall high telehealth satisfaction. General self-efficacy and attitudes toward technology predicted telehealth satisfaction, with no sex differences. Patients used a variety of technology and were moderately confident with it, suggesting an opportunity to expand virtual care beyond the telephone.

## Abbreviations

AF: atrial fibrillation
HTSE: health-technology self-efficacy
MCAR: missing completely at random
TSQ: telehealth satisfaction questionnaire
